# Immune checkpoint inhibition in undifferentiated cervical sarcoma with early pelvic recurrence: a case report

**DOI:** 10.3389/fonc.2026.1783604

**Published:** 2026-04-21

**Authors:** Yazhu Li, Jing Li, Huifang Liu, Qiting Tang, Limei Wang

**Affiliations:** 1 Department of Gynecology, The Seventh Medical Center of Chinese PLA General Hospital, Beijing, China; 2 Department of Pathology, The Seventh Medical Center of Chinese PLA General Hospital, Beijing, China

**Keywords:** immune checkpoint inhibitor, PD-L1, recurrent gynecologic malignancy, toripalimab, undifferentiated cervical sarcoma

## Abstract

**Background:**

Undifferentiated cervical sarcoma is an extremely rare and highly aggressive malignant mesenchymal tumor. It is associated with a poor prognosis, limited responsiveness to conventional chemotherapy and radiotherapy, and a lack of standardized treatment strategies.

**Case presentation:**

We report a 56-year-old postmenopausal woman who presented with vaginal bleeding and was diagnosed with International Federation of Gynecology and Obstetrics (FIGO) stage IIB undifferentiated cervical sarcoma. She underwent a modified laparoscopic radical hysterectomy with bilateral salpingo-oophorectomy, and gross intraoperative findings confirmed parametrial involvement. Postoperatively, she received three cycles of adjuvant chemotherapy. However, two months after surgery, during chemotherapy, she developed vaginal cuff bleeding and pelvic recurrence. Immunohistochemical analysis demonstrated programmed death-ligand 1 (PD-L1) positivity. The patient was subsequently treated with toripalimab monotherapy for six cycles, achieving complete tumor regression and a clinical complete response. This was followed by four cycles of consolidation immunotherapy. At the most recent follow-up, the patient remained disease-free.

**Conclusion:**

This case highlights the diagnostic challenges and aggressive clinical behavior of undifferentiated cervical sarcoma and suggests a potential role for immune checkpoint inhibition in PD-L1-positive recurrent disease. Further studies are needed to confirm the findings.

## Introduction

Cervical sarcoma is an exceptionally rare malignant tumor of the uterine cervix, accounting for less than 1% of all cervical malignancies (1). Among its histological subtypes, undifferentiated sarcoma is a diagnosis of exclusion, defined by the absence of specific morphological or immunophenotypic differentiation (2). Undifferentiated cervical sarcoma, as a distinct entity within this category, is particularly uncommon. Owing to its extreme rarity, independent clinical studies are scarce. Consequently, standardized treatment guidelines and consensus recommendations are lacking. In this report, we describe a case of undifferentiated cervical sarcoma with early pelvic recurrence after postoperative chemotherapy, in which immunotherapy monotherapy achieved a remarkable therapeutic response.

## Case presentation

### Chief complaints

A 56-year-old woman, married and nulliparous, was admitted to our hospital with a chief complaint of vaginal bleeding for half a month after 9 years of menopause.

### History of present illness

Four to five months prior to admission, she developed watery vaginal discharge without obvious inducement, odor, or discomfort. Approximately two weeks before admission, she experienced daily vaginal bleeding of a small volume and bright red color. Five days prior to admission, she was evaluated at a local hospital, where a cervical biopsy suggested a malignant tumor. Pelvic ultrasonography revealed minimal intrauterine fluid and a solid cervical mass, and she was referred to our institution for further management. The patient had no significant past medical or surgical history and no relevant family history.

### Physical examination

Physical examination showed stable vital signs. Gynecological examination revealed loss of normal cervical morphology, with an exophytic, cauliflower-like cervical mass of approximately 5 cm in diameter. The mass was friable and malodorous, with invasion of the vaginal fornix. Rectovaginal examination demonstrated thickening of the right parametrium without extension to the pelvic wall, and the rectal mucosa was smooth.

### Laboratory examinations and preoperative pathological examinations

Laboratory tests showed a fasting blood glucose level of 7.2 mmol/L, and serum tumor markers were within normal ranges. Preoperative cervical biopsy demonstrated a malignant mesenchymal tumor with marked nuclear atypia and frequent mitoses. The immunohistochemistry findings were as follows: p16 (-), vimentin (+), Friend leukemia integration 1 (FLI-1; +), CD31 (partial +), pan-cytokeratin (weak +), desmin (-), integrase interactor 1 (+), Brahma-related gene 1 (+), S-100 (-), smooth muscle actin (SMA; partial +), melan-A (-), and human melanoma black-45 (-), suggesting the possibility of epithelioid angiosarcoma.

### Imaging examinations

**Figure 1:**
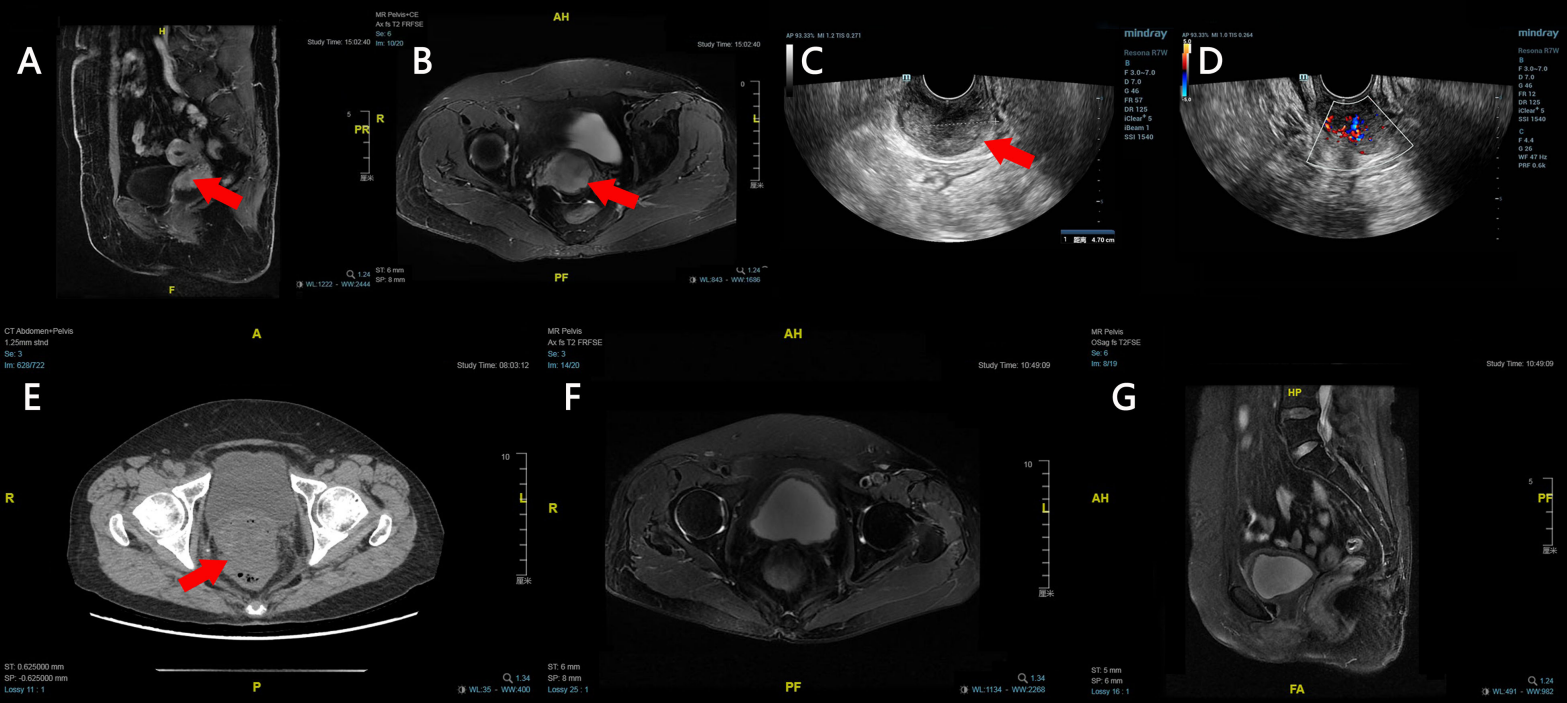
Imaging findings before and after treatment for cervical sarcoma. **(A, B)** Preoperative pelvic magnetic resonance imaging demonstrates a solid mass (red arrows) within the cervix. **(C, D)** Preoperative transvaginal ultrasound reveals a solid cervical mass measuring 4.7 cm × 4.2 cm × 2.8 cm (red arrow), with rich internal blood flow signals on color Doppler. **(E)** A pelvic computed tomography scan obtained two months postoperatively showed a new mass (red arrow, 5.22 cm × 4.8 cm) located between the bladder and rectum, indicating disease progression. **(F, G)** Pelvic magnetic resonance imaging after six cycles of immunotherapy shows no evidence of a residual or recurrent mass in the pelvic cavity. **(A)** sagittal LAVA Flex T1 fat-suppressed contrast-enhanced image; **(B)** axial FS T2 FRFSE sequence; **(F)** axial FS T2 FRFSE sequence; **(G)** sagittal FS T2 FSE sequence.

Pelvic ultrasound revealed a hypervascular solid cervical lesion measuring approximately 4.7 cm. Contrast-enhanced pelvic magnetic resonance imaging demonstrated an enlarged cervix with a heterogeneously enhancing mass (approximately 4.6 cm × 3.9 cm × 1.5 cm) showing high signal intensity on diffusion-weighted imaging, consistent with a malignant cervical tumor (**Figures 1A–D**). Chest and abdominal computed tomography scans showed no definite distant metastases.

### Postoperative pathological examinations, diagnosis, and treatments

**Figure 2:**
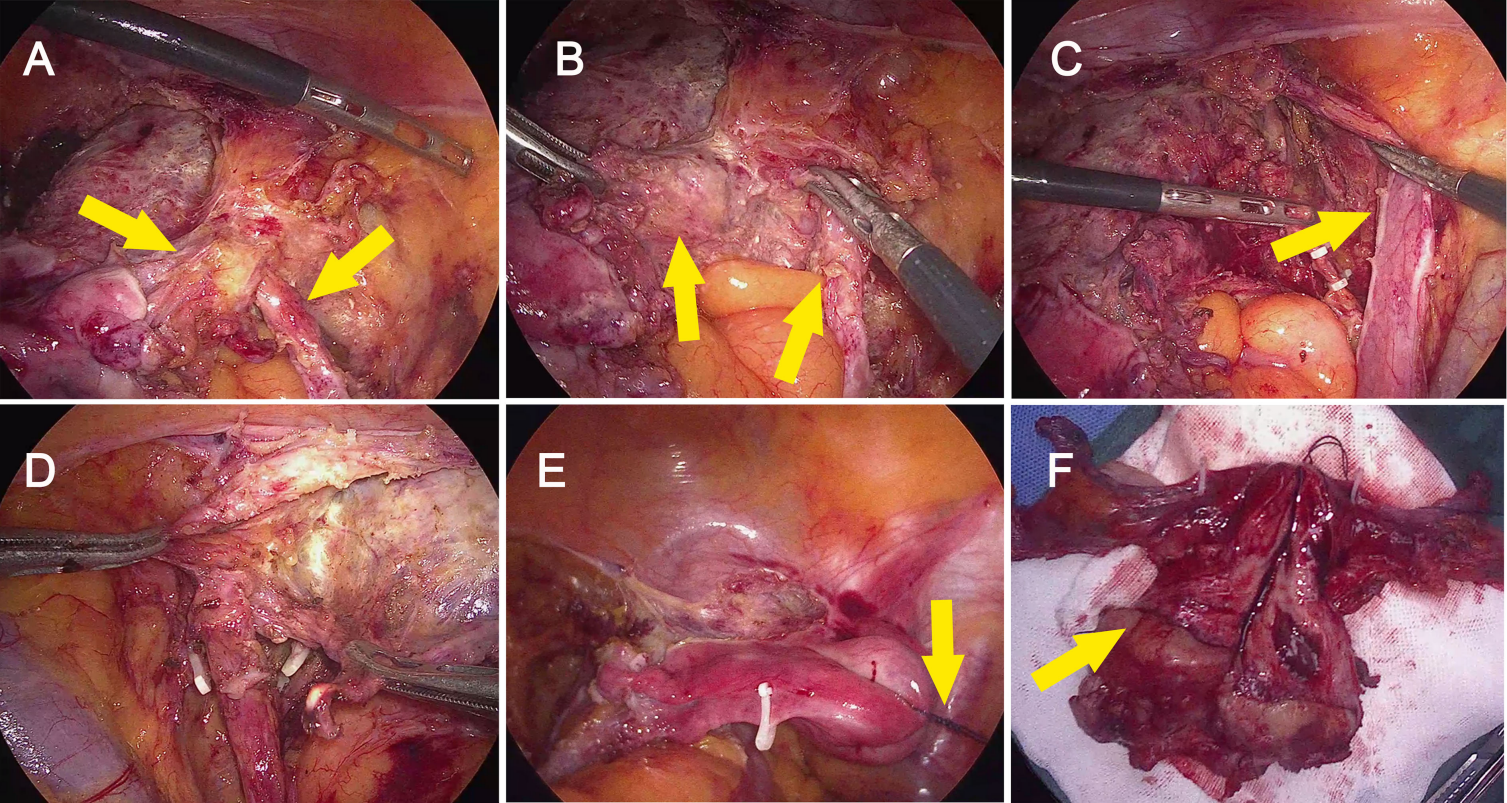
Intraoperative findings and gross specimen of cervical sarcoma. **(A)** Intraoperative view showing tumor infiltration of the right parametrium and close adhesion to the ureter (yellow arrows). **(B)** Dissection of the right ureter to expose the metastatic lesion within the right parametrium (yellow arrows). **(C)** Complete mobilization of the right ureter, allowing for resection of the parametrial metastatic lesion (yellow arrow). **(D)** Intraoperative view of the left parametrium, which was free of tumor infiltration. **(E)** Surgical field illustrating the use of a suture suspension technique (yellow arrow) to elevate the uterus, replacing a conventional uterine manipulator. **(F)** Gross specimen of the hysterectomy with bilateral adnexectomy, revealing a large volume of tumor tissue in the cervix.

**Figure 3:**
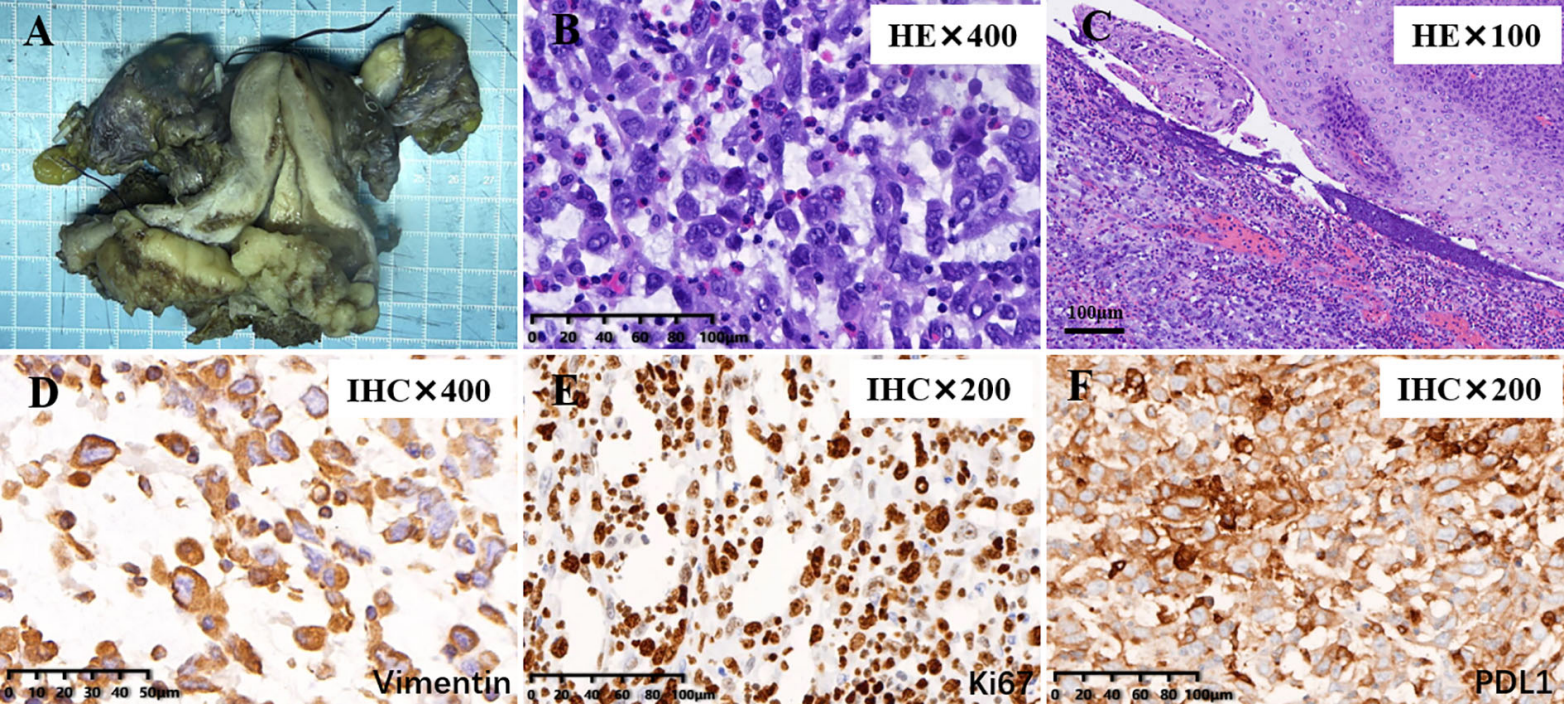
Pathological and immunohistochemical findings of the cervical sarcoma. **(A)** Gross specimen from hysterectomy with bilateral adnexectomy showing an exophytic cervical tumor protruding into and invading the full thickness of the cervical canal wall. A metastatic nodule is present in the extra-serosal adipose tissue at the right cervico-uterine junction. **(B)** Hematoxylin and eosin staining (H&E, ×400) reveals singly scattered tumor cells with marked cytological atypia and readily identifiable mitotic figures. **(C)** H&E (×100) confirms tumor involvement of the vaginal fornix. **(D)** Immunohistochemical staining for vimentin (×200) shows diffuse vimentin positivity in tumor cells, supporting a diagnosis of mesenchymal-origin undifferentiated sarcoma. **(E)** Ki-67 immunohistochemistry (×200) demonstrates a high proliferative index (up to 70%) in tumor cells. **(F)** PD-L1 immunohistochemistry (×400) reveals strong positive expression in tumor cells, with a combined positive score > 80.

Because the preoperative impression was a malignant mesenchymal tumor, a modified laparoscopic approach was selected. To reduce the risk of tumor spillage, the uterus was suspended without the use of a uterine manipulator, a sufficiently long vaginal cuff was resected to ensure an adequate surgical margin, and the vagina was closed intraoperatively. Intraoperatively, the tumor was confirmed to have penetrated the uterine serosa and extended into the surrounding tissues, with invasion of the right parametrium and close involvement of the right ureter, indicating parametrial spread (**Figures 2A–F**). Postoperative pathological examination revealed a malignant mesenchymal tumor involving the cervix from the 9 to 1 o’clock position, measuring 6 cm × 3 cm × 2 cm, with full-thickness invasion of the cervical wall. A metastatic tumor nodule (1.2 cm × 1.0 cm × 1.0 cm) was identified in the extra-serosal adipose tissue at the cervico-uterine junction. Perineural invasion and intravascular tumor emboli were present (**Figure 3A**). The immunohistochemistry findings were as follows: vaginal wall margin (-4), p16 (-), pan-cytokeratin (partial weak +), vimentin (weak +), p40 (partial +), CD31 (scattered +), factor VIII (focal +), Ki-67 (+ ~60% in dense areas), CD34 (-; background vessels +), D2-40 (-; background lymphatics +), FLI-1 (-), SMA (+/-), desmin (-), h-caldesmon (-), CD10 (weak +), estrogen receptor (-), progesterone receptor (-), cyclin D1 (-), and programmed death-ligand 1 (PD-L1) (+, combined positive score>80) (**Figures 3B–F**). PD-L1 expression was assessed using immunohistochemistry with the VENTANA platform and the PD-L1 (SP263) assay (Roche Diagnostics). Immunostaining was performed using the EnVision two-step method.

The final diagnosis was undifferentiated cervical sarcoma, International Federation of Gynecology and Obstetrics (FIGO) stage IIB. During hospitalization, the patient was newly diagnosed with type 2 diabetes mellitus. Adjuvant chemotherapy with doxorubicin (95 mg intravenously) was administered postoperatively for three cycles.

### Early pelvic recurrence and subsequent immunotherapy

Approximately two months after surgery, following three cycles of adjuvant chemotherapy, the patient developed massive vaginal bleeding and required urgent clinical evaluation. Because immediate confirmation of local recurrence and hemostatic management were prioritized in this emergency setting, gynecological examination and pelvic computed tomography were performed first. These examinations revealed a vaginal stump mass located between the bladder and rectum measuring 5.2 cm × 4.8 cm (**Figure 1E**). A repeat biopsy of the lesion also supported early pelvic recurrent undifferentiated sarcoma. Emergency transcatheter embolization of the internal iliac artery was performed for hemostasis. Following stabilization, given the rapid postoperative recurrence, chemotherapy failure, and high PD-L1 expression, and the absence of a clearly effective standard salvage treatment, toripalimab was initiated after informed consent was obtained; it was also financially accessible to the patient. The patient received toripalimab monotherapy (240 mg every three weeks). After three cycles, vaginal bleeding ceased, and imaging demonstrated marked tumor regression. Following six cycles of immunotherapy, pelvic imaging confirmed the complete disappearance of the lesion, consistent with a clinical complete response (**Figures 1F, G**). During toripalimab treatment, the patient developed marked hyperglycemia, leading to a temporary interruption of immunotherapy for two months. The patient had already been diagnosed with type 2 diabetes mellitus, but adherence to diabetic management was poor, and glucose-lowering medication was not taken, resulting in inadequate glycemic control. Therefore, this event was considered more likely related to underlying diabetes, although a treatment-related contribution could not be completely excluded. Because C-peptide testing and formal endocrinological evaluation were not performed, a definite endocrine immune-related adverse event could not be established. After endocrinology-directed metabolic stabilization, toripalimab was resumed, followed by four cycles of consolidation immunotherapy. No other definite immune-related adverse events were observed. A longer course had been recommended, but the patient did not complete the planned 1-year treatment because of suboptimal adherence.

### Follow-up

At follow-up, gynecological examination and pelvic imaging revealed no signs of local pelvic recurrence or distant metastasis. Additionally, the patient’s resumption of previous work activities indicates both effective disease control and significant functional recovery following immune checkpoint inhibition.

**Figure 4:**
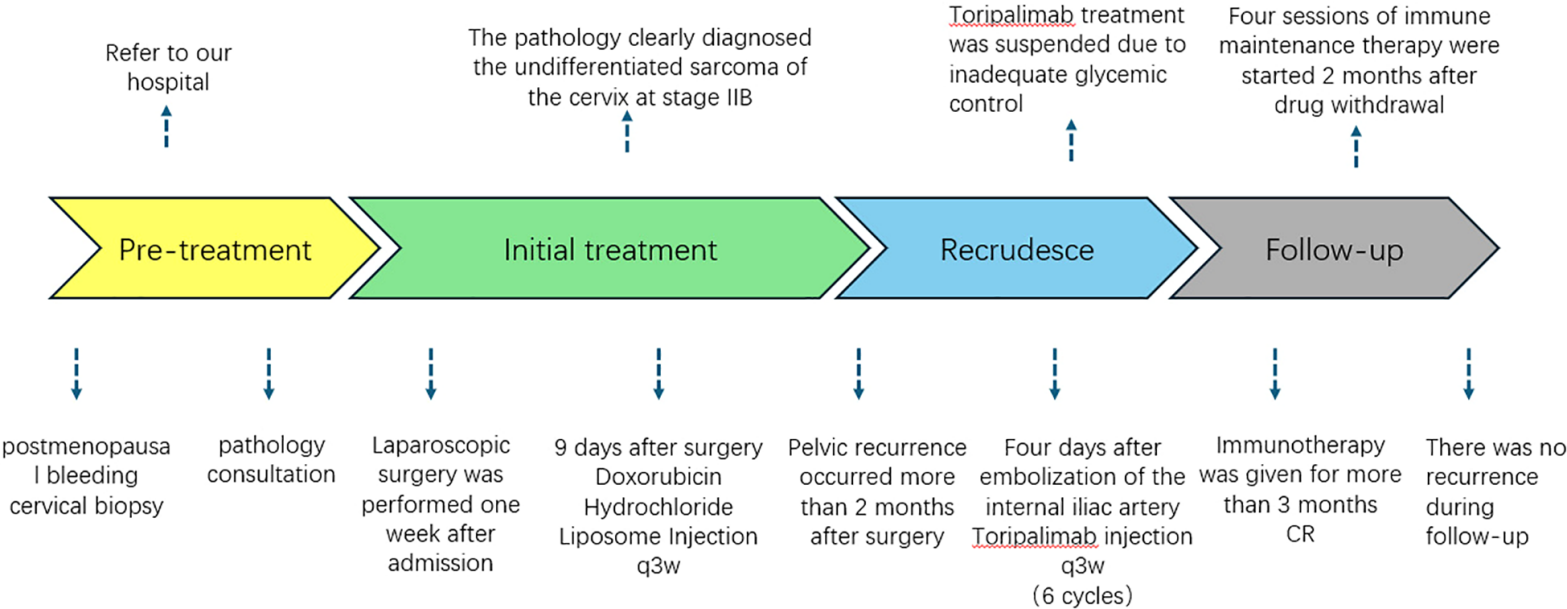
Timeline of the patient’s diagnosis and treatment.

To provide a clear overview of the diagnostic and therapeutic course for this patient with FIGO stage IIB undifferentiated cervical sarcoma, we developed a detailed timeline of clinical management (**Figure 4**).

## Discussion

Undifferentiated cervical sarcoma is an exceptionally rare malignancy, with available evidence largely limited to isolated case reports and small retrospective series (3, 4). Owing to its rarity and histological heterogeneity, the diagnosis and treatment of this malignant tumor remain challenging. The present case is notable for its cervical origin, aggressive pathological features, early postoperative pelvic recurrence, and subsequent disease control achieved with immune checkpoint inhibition, highlighting several clinically relevant issues. Clinically, patients with undifferentiated cervical sarcoma often present with nonspecific symptoms such as postmenopausal bleeding or abnormal vaginal discharge, which are symptoms indistinguishable from those of more common cervical cancers (5). Histologically, undifferentiated sarcomas lack specific differentiation and are characterized by marked nuclear atypia and high mitotic activity (6). Negative desmin and h-caldesmon also argued against leiomyosarcoma. Overall, the diagnosis of undifferentiated cervical sarcoma was established through integrated evaluation of morphologic features, comprehensive immunohistochemical findings, and the clinical context. These features contribute to significant diagnostic difficulty, particularly in small biopsy specimens. In the present case, focal epithelioid morphology and partial expression of endothelial markers in the biopsy specimen initially suggested epithelioid angiosarcoma. However, focal or aberrant expression of lineage markers is well recognized in undifferentiated sarcomas and should be interpreted with caution, particularly in limited samples (7). The absence of diffuse endothelial marker expression and definitive vasoformative structures in the resection specimen argued against angiosarcoma (8). Although partial p40 positivity raised the possibility of sarcomatoid squamous cell carcinoma, no distinct epithelial component or conventional squamous differentiation was identified morphologically. Moreover, pan-cytokeratin expression was only focal and weak, and p16 was negative, making a typical HPV-associated cervical squamous carcinoma less likely. Because focal cytokeratin expression was present, carcinosarcoma could not be entirely excluded on immunophenotypic grounds alone; however, the absence of a distinct epithelial component meant that this diagnosis was not favored. Negative desmin and h-caldesmon also argued against leiomyosarcoma. Overall, the diagnosis of undifferentiated cervical sarcoma was established through integrated evaluation of morphologic features, comprehensive immunohistochemical findings, and the clinical context.

Owing to the rarity of undifferentiated cervical sarcoma, no standardized treatment guidelines exist. Although the LACC trial raised concerns about minimally invasive surgery for cervical carcinoma, its applicability to primary cervical sarcoma remains uncertain. Surgical resection followed by adjuvant therapy is the most commonly adopted strategy and is generally recommended for cervical sarcomas with high-risk features (9, 10). Given the presence of multiple high-risk pathological features in our patient, adjuvant systemic chemotherapy was administered after surgery. Anthracycline-based regimens, particularly doxorubicin, are widely used as the first-line therapy for high-grade uterine and soft tissue sarcomas (11). In this context, adjuvant single-agent doxorubicin was considered an acceptable systemic option for postoperative treatment. Although combination chemotherapy may improve response rates in selected settings, its survival advantage over single-agent doxorubicin has not been clearly established. However, a previous study showed that undifferentiated sarcoma often exhibits limited sensitivity to conventional cytotoxic chemotherapy, with high rates of early recurrence (12). Consistent with these observations, our patient experienced rapid pelvic recurrence shortly after completion of adjuvant doxorubicin-based chemotherapy. Therefore, it cannot be concluded that the early recurrence was solely attributable to the use of single-agent chemotherapy; rather, the early relapse despite appropriate surgical resection and standard systemic therapy more likely reflects the intrinsic chemoresistance and aggressive biological behavior of this tumor subtype.

The early relapse despite appropriate surgical resection and standard systemic therapy reflects the intrinsic chemoresistance and aggressive biological behavior of this tumor subtype, rather than suboptimal treatment selection. The decision to initiate PD-1 blockade in this case was based on both the clinical presentation and underlying tumor biology. Undifferentiated sarcomas are characterized by profound loss of lineage differentiation and high genomic instability, which may increase neoantigen burden and enhance tumor immunogenicity (2, 13). In our patient, a high proliferative index (Ki-67 of ~60%) further indicated an aggressive tumor biology and a rapid cell turnover, features that have been associated with increased immune recognition in certain malignancies. Clinically, early recurrence and the lack of response to anthracycline-based chemotherapy placed this patient within a population increasingly considered appropriate for immunotherapy exploration. Emerging evidence from clinical trials and case series suggests that patients with certain high-grade and undifferentiated sarcomas may benefit from PD-1 blockade, despite modest overall response rates (10, 14). Although data on immunotherapy in undifferentiated cervical sarcoma are extremely limited, our patient achieved favorable local disease control after toripalimab treatment. Notably, the tumor exhibited positive PD-L1 expression, supporting the potential role of immune checkpoint inhibition as a salvage option in chemotherapy-refractory patients.

Several limitations of this study should be acknowledged. First, this was a single-case observation, and the findings cannot be generalized to all patients with undifferentiated cervical sarcoma. Second, the follow-up duration was relatively short, and a longer observation period is required to determine the durability of disease control and overall survival benefit. Third, repeat laboratory and imaging assessment was not performed in a standardized manner after the second cycle of adjuvant chemotherapy, and a more comprehensive restaging evaluation, including assessment beyond the pelvis, was not completed before recurrence was confirmed. Because the patient presented with acute massive vaginal bleeding, urgent pelvic-focused evaluation was prioritized to guide immediate hemostatic management. This may have limited the completeness of disease reassessment at the time of early recurrence. Fourth, a formal multidisciplinary team discussion was not conducted during the diagnostic and therapeutic course. Clinical decisions were primarily made by the gynecologic oncology team based on the available pathological findings, imaging results, and the patient’s clinical condition. The absence of formal multidisciplinary input may have limited the comprehensiveness of disease assessment and treatment planning. In addition, some ancillary immunohistochemical markers, including Claudin-4 and SOX10, were not available, which limited the further exclusion of epithelial origin and malignant melanoma.

In conclusion, this case underscores the diagnostic complexity and aggressive clinical behavior of undifferentiated cervical sarcoma. Accurate diagnosis requires integration of morphology, extensive immunohistochemistry, and clinical correlation. Conventional chemotherapy may be insufficient, and immunotherapy may represent a promising salvage option. Further accumulation of clinical and molecular data is needed to refine diagnostic criteria and optimize treatment strategies for this rare malignancy.

## Data Availability

The original contributions presented in the study are included in the article/supplementary material. Further inquiries can be directed to the corresponding author.
